# Application of a screening tool to understand the medication habits of patients with swallowing difficulty: a prospective observational study

**DOI:** 10.1007/s11096-025-01901-7

**Published:** 2025-03-27

**Authors:** A. Harnett, L. J. Sahm, E. Burke, D. Lyons, S. Byrne

**Affiliations:** 1 Pharmaceutical Care Research Group, School of Pharmacy, University College Cork, Cork, Ireland; 2 University Hospital Limerick, Dooradoyle, Limerick, Ireland; 3 Pharmacy Department, Mercy University Hospital, Grenville Place, Cork, Ireland

**Keywords:** Dysphagia, Hospital inpatient, Medication modification, Solid oral dosage form, Swallowing difficulty, SWAMECO

## Abstract

**Background:**

Difficulty swallowing solid oral dose forms can result in non-adherence and thus can negatively impact patient outcomes. It can be challenging for healthcare professionals to readily identify patients who present with this difficulty within the hospital setting.

**Aim:**

To apply the “Swallowing Difficulties with Medication Intake and Coping Strategies” (SWAMECO) questionnaire at admission to hospital to elucidate the medication habits of patients when taking medication at home.

**Method:**

This prospective observational study took place in acute urban teaching hospital in Ireland in July 2023. Eligible adults completed the SWAMECO, describing their difficulty and the coping strategies used. Data on age, sex, medicines and disease states, as per International Classification of Diseases and Related Health problems (ICD-10) were collected.

**Results:**

Self-reported prevalence of swallowing difficulties was 10% (41/409) of whom, 14 patients (median age; 67 years (range 40–86 years), 78.6%; female) completed the SWAMECO. The majority reported having a sensation of medication getting stuck in the pharynx. Swallowing difficulty caused anxiety for some patients and contributed to non-adherence. The most common coping strategy was splitting tablets. Paracetamol was the most frequent solid oral dose form associated with swallowing difficulty and hypertension was the most diagnosed condition. Alternative formulations were available for over half (56%) of the solid oral dose forms prescribed.

**Conclusion:**

The SWAMECO can provide valuable information by identification of patients with swallowing difficulty. This may facilitate clinical pharmacist intervention to ensure safe administration of oral medicines and to enhance patient adherence by providing tailored solutions.

**Supplementary Information:**

The online version contains supplementary material available at 10.1007/s11096-025-01901-7.

## Impact statements

- Screening with SWAMECO identified patients with difficulty swallowing medicines and, if performed upon admission, would facilitate referral to a clinical pharmacist for medicines optimisation.
- Knowledge of modifications undertaken e.g. splitting tablets, can mitigate the potential risks of inappropriate alterations of solid oral dosage forms.
- Clinically appropriate substitution of solid oral dosage forms with alternative formulations may positively affect medication adherence.

## Introduction

Whilst tablets and capsules present a convenient way to administer medicines, some patients experience difficulty swallowing these solid oral dose forms [1]. When the physiological swallowing process is dysfunctional a diagnosis of dysphagia may be made [2], however a difficulty swallowing solid oral dose forms can occur in the absence of any formal diagnosis [3]. The latter can be described as a functional dysphagia or as pill aversion [3]. If a patient considers that they have a difficulty swallowing these medicines, then the criteria for having a difficulty swallowing are met. A recent study reported a prevalence of swallowing difficulty of 9.6% in acute hospital inpatients without an enteral feeding tube with the majority having no formal diagnosis of dysphagia [4]. A number of factors can negatively affect the swallowability of solid oral dose forms including size, shape and surface characteristics, with colour, sensation in the mouth, and taste also reported [5–7].

Patients in hospital with difficulty swallowing solid oral dose forms, are potentially at risk of medication administration errors which may cause harm [8–16]. The error is often associated with the requirement to modify this medication to facilitate administration, most commonly by crushing [1]. However, any alteration prior to administration is considered a modification [17] and whilst this provides a practical solution, there may be unintended consequences. Those involved in administering, and potentially crushing of, medicines, conventionally nursing personnel in the Irish hospital system, may be exposed to aerosolised cytotoxic, teratogenic or hormonal particles [18] and the resulting substance may also irritate airways or skin [18]. Additionally, the destruction of galenic formulation, which falls outside of the product authorisation [19], may affect the pharmacokinetic profile e.g. in the case of sustained release medications, with resultant dose dumping and toxicity [18, 20] and/or therapeutic failure. Those involved in these modifications, whether healthcare professional or patient, may not be aware of these risks or may see them as necessary [21, 22]. Difficulty swallowing can also contribute to intentional non-adherence with negative patient consequences [3]. To mitigate the risk to patients a proactive approach to identify swallowing difficulties should be taken, as strategies can then be put in place to help these patients [23]. This is especially crucial for the older adult (65 years or older), given the current trends worldwide [24], showing that older people experience more co-morbidities resulting in more prescribed medications [25]. Coupled with this are the specific age-related changes including physiological function that negatively impacts swallow [26, 27]. Thus early (in the admission process) identification, by healthcare professionals, may allow for a range of solutions being offered including; change of formulation, use of an alternative route for administration, and /or advice on safe modification of the solid oral dose form.

One means of identifying hospital patients with difficulty swallowing solid oral dose forms is to screen patients. A team of Swiss pharmacists developed and validated the **SWA**llowing difficulties with **ME**dication intake and **CO**ping strategies (SWAMECO) questionnaire, hereafter SWAMECO, in patients suffering from systemic sclerosis [28]. SWAMECO was subsequently validated in community-dwelling adult patients [29]. The SWAMECO consists of 18 items and takes approximately 5 minutes to complete. It also encompasses a free text section to capture patients’ comments (See electronic supplementary material ‘X’).

### Aim

To use the SWAMECO to elucidate the medication habits of patients with swallowing difficulties when taking medicines at home.

### Ethics approval

Ethics approval was obtained from the Research Ethics Committee, University Hospital Limerick (REC 057/2023), May 2023.

## Method

### Study design

This is a prospective interventional study with the SWAMECO completed by hospital patients who have reported a difficulty with swallowing solid oral dose forms.

Ten of the 18 SWAMECO items (Items 1, 2, 3, 4, 11, 13, 14, 15, 16, 18) have a binary outcome, yes/no with one item providing yes/no/not applicable options (Item 10). A further three items give options to select an answer (Items 6, 9, 12) with two of those Items also providing a free text section (Items 6, 9). Item 7 comprises a visual analogue scale (0 = no effect and 10 = extreme effect), and item 5 requires the patient to put an ‘X’ on a diagram. The remaining two items require free text answers (Items 8, 17).

### Time period for data collection

The SWAMECO data were collected from Monday 3rd July to Monday 10th July 2023.

### Study setting

The study was conducted in a large teaching hospital in the Mid-west of Ireland with a catchment area of circa 410,000 people, an intensive care unit and a 24-hour emergency department admitting undifferentiated acute medical and surgical patients [30, 31].

### Inclusion criteria

(i) Patients prescribed solid oral dose forms and had a difficulty swallowing them (ii) 18 years or more and (iii) An inpatient of the hospital at 08:00 h on the day of data collection.

### Exclusion criteria

(i) Patients unable to provide written informed consent e.g. patients with acute or chronic confusional state. (ii) Patients in critical care, on coronary care wards, on cancer services ward, paediatric wards or psychiatric unit. (iii) Outpatients and day cases. (iv) Patients receiving all their prescribed medicines via an enteral feeding tube.

### Data collection

Patients were identified from a ward census generated daily. All patients were interviewed by a research assistant (EB) to establish their swallowing status. For any patient with a difficulty swallowing solid oral dose forms the study was explained, and a participant information leaflet provided. If agreeable and inclusion criteria were met, the patient provided written informed consent.

Patients completed the SWAMECO. EB remained with the patient while completing the SWAMECO, to answer any questions. Data on age, sex, medicines (name, dose, strength) and disease states, as per International Classification of Diseases and Related Health problems (ICD-10) [32], were collected.

Data were collected in hardcopy, and then transferred onto a Microsoft Excel spreadsheet (IBM Corp. version 2403). All data were stored securely with restricted access and in full compliance with General Data Protection Regulations [33].

### Data analysis

Data were analysed using Microsoft Excel® (2017). Descriptive statistics were calculated for participants age, sex, number and name of medicines taken and diagnosed medical conditions. A median and range was reported for age as the data were not normally distributed. Disease states were classified according to the ICD-10 [32].

Descriptive statistics were also reported for participant responses to the 18 items which are presented as percentages and frequency. For the response to Item 7, using the visual analogue scale, the ‘X’ placed by the patient on the scale was translated to the nearest whole number using a ruler to measure the distance of the ‘X’ from zero.

The availability of an alternative oral formulation, such as a liquid or orodispersible tablet, or the availability of a therapeutic alternative in the same class, which was available as a liquid or orodispersible tablet was established by checking the electronic medicine catalogue in the hospital pharmacy.

## Results

**Fig. 1:**
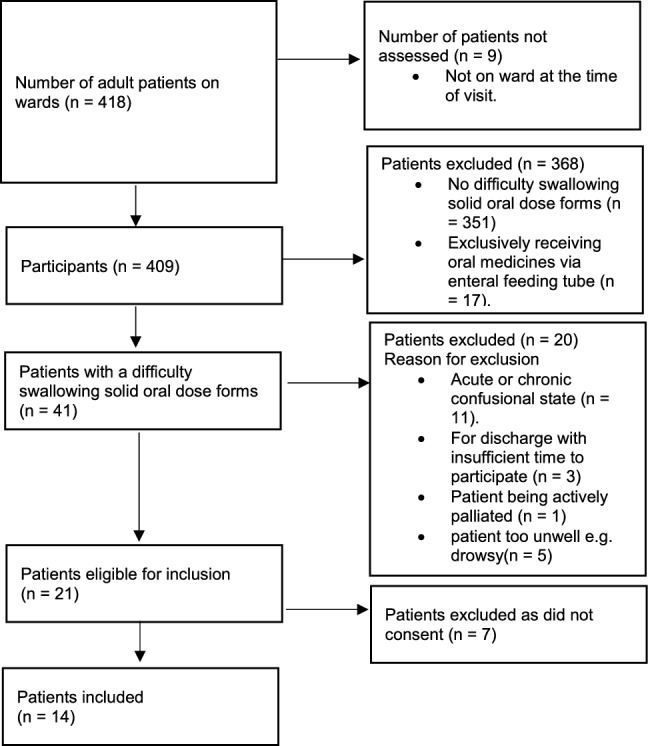
Flow diagram of patient selection

Of 418 inpatients, 14 completed the SWAMECO (Fig. 1).

The median age of participants (n=14) was 67 years (range 40–86 years) and 78.6% were female. A total of 56 different diagnosed conditions were recorded in these patients. (Table 1).

**Table 1:** Demographic details of the patients identified with swallowing difficulty including the patient-identified location of the swallowing difficulty (Items 5 of SWAMECO)

Participant demographics	Number of patients n=14
Median age	67 years (range 40–86)
Gender	Female 78.6% / Male 21.4%
Mean number of medications per patient	16.9 (range 15–21)
Number of patients with diagnosis of dysphagia	0

Frequent diagnosis recorded	
Hypertension	6 patients
Hypothyroidism	5 patients
Hypercholesterolemia	4 patients
Diverticular disease	3 patients
Type II diabetes mellitus (T2DM)	3 patients

Location identified on human profile	Number of patients selecting the area (Percent) n=14 patients
Tongue	0
Mouth	2 (14.3%)
Throat	1 (7.1%)
Pharynx	11 (78.6%)
Oesophagus	3 (21.4%)
Stomach	0

In total 237 medicines were prescribed for these 14 patients (mean 16.9 per patient, range: 15–21). Of these, three quarters (176 prescriptions) were prescribed for the oral route, with 63.3% (150 prescriptions) being solid oral dose forms. Alternative formulations (liquid, oro-dispersible tablets) were available for 56.0% (84/150) of those prescriptions. An alternative liquid or orodispersible formulation in the same therapeutic class was available for a further 12.0% (18/150).

### Description of the swallowing difficulty

**Fig. 2:**
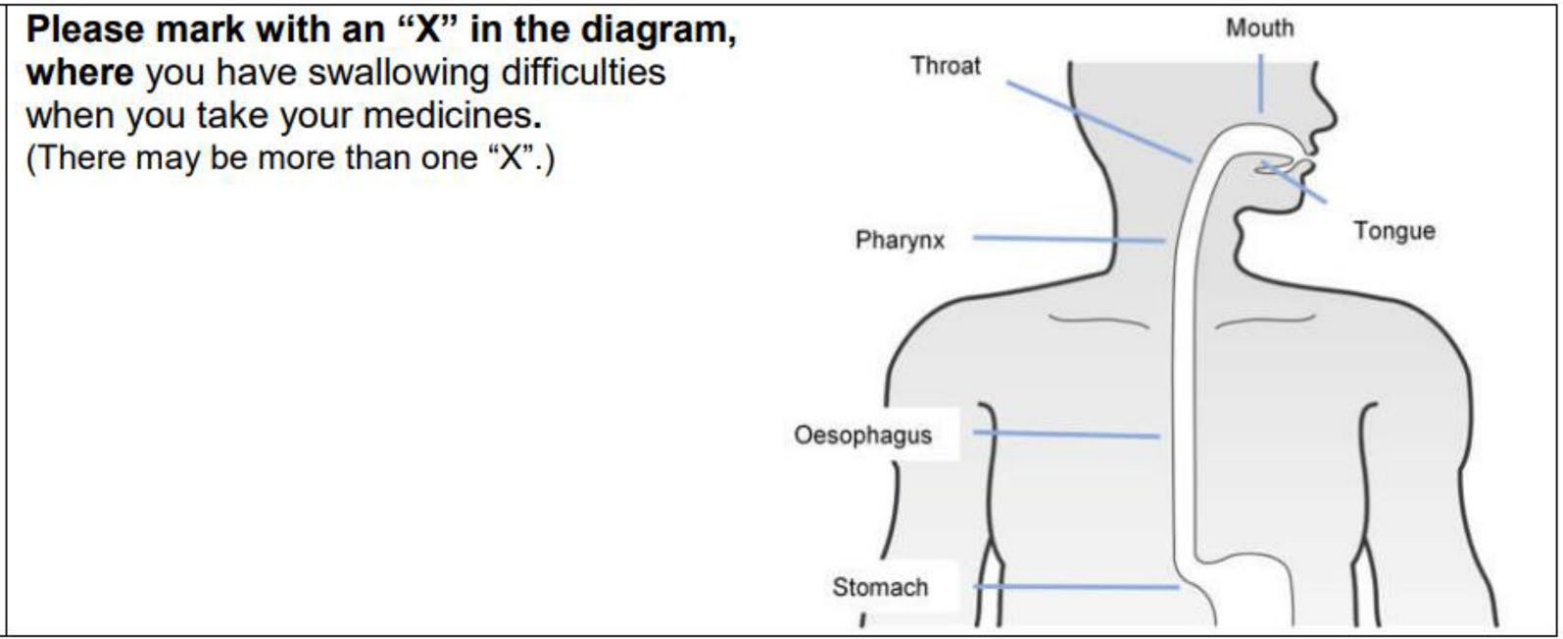
Item 5 on the Swallowing difficulties with Medication intake and Coping strategies (SWAMECO) questionnaire

Patients were asked to identify an area associated with the swallowing difficulty (Item 5) (Fig. 2). A total of 17 locations were identified by the 14 patients. Three patients reported more than one location (median 1, range 1–2). The most common location that patients identified was the pharynx (Table 1).

Most commonly patients reported a feeling of the medication getting stuck when swallowing (Item 6) with 85.7% citing this as their experience when swallowing medicines (Table 1). Over half of the patients (57.1%) reported having a fit of coughing when swallowing solid oral medicines.

### Effect of the swallowing difficulty

**Fig. 3:**
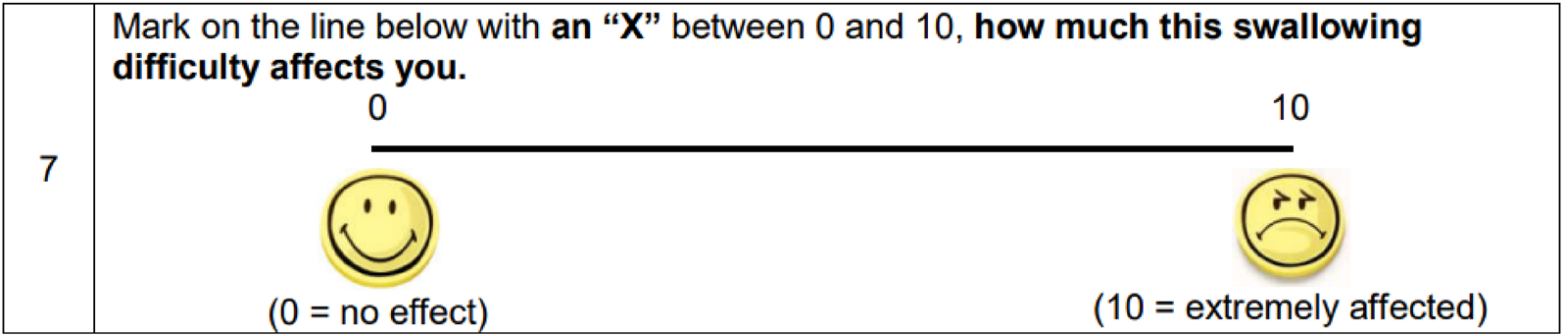
Item 7 on the on the swallowing difficulties with medication intake and coping strategies (SWAMECO) questionnaire

Nine patients (64.3%) declared that they worried about their swallowing difficulties (Item 2) and 8 (57.1%) agreed that they were afraid of the next time that they had to take medication (Item 3). Patients were also asked to rate (on a scale of 0–10) how much they were affected by their swallowing difficulty (Item 7) (Fig. 3).

In total, 11 (78.6 %) of participants reported a value ≥ 5 (Supplemental Table 1). All patients reported being affected by their swallowing difficulty with a median score of 6 (range 2–10).

### Medicines identified as associated with swallowing difficulty

Patients reported several different medicines associated with swallowing difficulty (Item 8) , the most common of which was paracetamol (Supplemental Table 2).

### Coping strategies

When asked whether they used any modification to aid taking their medications (Item 9), seven patients (50.0%) declared that they did not. Splitting tablets was the most common method of modification (n = 6) and some patients reported more than one strategy. Crushing (n = 3), chewing tablets (n = 2) and dispersing tablets in a liquid (n = 1) were also reported. Of those who modified their medication, three (42.9%) had spoken to their doctor or pharmacist about this modification (Item 10).

Seven patients (50.0%) reported that their head was in a straight position when they swallowed their medicines with three (21.4%) reporting that their head was tilted forward and four (28.6%) reporting that their head was tilted back (Item 12).

Thirteen of the 14 patients reported drinking something to help swallow their medicines (Item 13) and seven reported eating something to help (Item 14).

### Medication adherence

Eleven of the 14 participants (78.6%) reported that they had not missed any dose of their medications in the last 30 days (Item 17). Three (21.4%) reported intentional non-adherence with one patient missing doses on 15 of the previous 30 days.

Four patients (28.6%) reported “ever” missing doses of their medication because of their swallowing difficulty (Item 18).

Five patients (35.7%) reported that their doctor was aware that they had trouble swallowing their medications (Item 4), one of whom reported receiving an alternative formulation instead of the original (Item 11). Three patients, who reported that their doctor was not aware of their swallowing difficulty, also reported receiving an alternative formulation of their medication from their doctor or pharmacist instead of capsules or tablets (Item 11).

Twelve of the 14 inpatients (85.7%) reported having a dry mouth during the day (Item 15) with eight (57%) reporting that they had dry eyes and/or nostrils (Item 16).

## Discussion

### Statement of key findings

This study reports the first use of the SWAMECO assessment in acute hospital inpatients. All participants self-completed the SWAMECO demonstrating that SWAMECO may be used to establish baseline information on patients ability to swallow solid oral dose forms. However, this self-completed SWAMECO was not suitable for all e.g. those with confusion. In this study the pharyngeal stage of swallowing was reported most frequently as causing the problem. Patients reported that swallowing difficulties caused worry, fear and in some cases negatively impacted adherence. Coping strategies included splitting and crushing tablets. Most prescriptions for solid oral dosage forms had an alternative formulation available.

### Strengths and weaknesses

This is the first study using the SWAMECO as a tool to better understand acute hospital patients’ difficulties with swallowing solid oral dose forms. All items were answered by all participants so a comprehensive analysis of the answers was possible. However, this study has some limitations. The sample size was small and conducted at a single site making it difficult to generalise the findings. The SWAMECO required informed written consent, thus patients unable to provide this consent were excluded as seen in other studies for example those with advanced dementia [34]. Additionally, as this is a self-reporting tool, subjectivity may have led to patients downplaying their swallowing difficulties and likely resulted in an underestimate of the prevalence of swallowing difficulties. Self-reporting of adherence has also been shown to be unreliable [35].

### Interpretation

In keeping with previous studies using SWAMECO in patients with systemic sclerosis [28] and in community-dwelling patients [29], the most common location identified as problematic was the pharynx. However, evidence suggests that patients are not always reliable in determining the location of the problem [36]. Nonetheless the addition of this information, whilst it may not be entirely accurate, is a mechanism by which the patient may express their lived experience and thus worthy of inclusion [37]. For the majority (87.5%), medicines getting stuck in the throat was the most common feeling reported by patients, which aligns with other studies [28, 29].

For two thirds of the participants, swallowing difficulties caused worry with approximately half reporting a fear of taking oral medicines as a result. This is slightly higher than reported in another study were half the patients reported worry and one fifth had a fear of taking oral medicines [28].

Patients reported using several techniques including, splitting, crushing and chewing solid oral dose forms and/or dispersing these in liquid, to facilitate swallowing. In most cases, this was done in the absence of advice from a healthcare professional. This finding is similar to other studies with SWAMECO [28, 29] and studies using other methodologies [47, 54]. This is concerning as modifying solid oral dose forms is not without risk or potential harm to the individual [18, 20, 38] . The use of the SWAMECO for adult patients at the point of admission to hospital or during the inpatient stay would help to identify (i) those with difficulties and (ii) their coping strategies, thus allowing clinical pharmacists to advise on appropriate prescribing of oral medicines, in collaboration with other members of the multidisciplinary team. In addition, pharmacists can offer guidance on the best strategies to use once the patient returns to the community setting. Whilst there are insufficient numbers of pharmacists working in Irish hospitals [39] the fact that the SWAMECO can be completed by the patient, may allow prioritisation for pharmacist review. An additional prioritisation strategy may include screening for medications known to cause gastrointestinal side effects e.g. xerostomia caused by antimuscarinic effects, cytotoxic damage to intestinal mucosa, or local irritation of the oesophagus by solid oral dose forms e.g. bisphosphonates [40, 41]. Medications that depress the central nervous system causing drowsiness are also reported as potentially causing dysphagia [40].

Bending the head forward with the head tilted slightly towards the chest has been reported to improve swallowing of medicines [42, 43]. However, only approximately 1 in 5 of the patients in our study had their head tilted slightly forward when taking their oral medication. This is higher than that reported by Messerli et al., [28] with a finding of approximately 1 in 10 of the patients with swallowing difficulty had their head tilted slightly forward. Thus, timely identification of these patients would offer an opportunity to counsel on an improved swallowing technique.

Approximately 1 in 5 of the participants reported intentional non-adherence in the last 30 days due to their swallowing difficulty, with one patient reporting intentional non-adherence approximately half of the month. In a study investigating medication adherence in those with swallowing difficulties in a general population, this figure increases to approximately 7 in 10 [44]. Non-adherence to medication can have significant health consequences [45] while good adherence is associated with positive health outcomes [46]. If clinical pharmacists knew about patients swallowing difficulties, they would be in a stronger position to recommend suitable alternative medications potentially aiding adherence.

Nine patients (64.3%) with difficulty swallowing, reported that their GP was not aware that they had trouble swallowing their medications, so it is hardly surprising that approximately two thirds of their medication had not been altered to ameliorate this. A study examining swallowing difficulties in community-dwelling patients found that pharmacists and doctors seldom enquired about swallowing difficulties and recommended that pharmacists adopt a more systematic approach to uncovering intentional non-adherence due to swallowing difficulties [47]. All patients met the definition for hyper-polypharmacy, defined as more than ten co-prescribed medications, placing the patients at increased risk of the negative consequences of polypharmacy including swallowing difficulty [48, 49].

Messerli et al., [28] proposed that dryness syndrome might offer an opportunity for counselling patients on medication. Their study, in community-dwelling patients with swallowing difficulties, found that two thirds reported dryness of the mouth, eyes and nose while our study found that the majority, greater than 85.0% reported dry mouth with over half reporting dry eyes and nostrils. Dry mouth is more commonly reported by females, older persons and those on certain medications [50–52]. Three quarters of our cohort were female with an average age of 65 years or more, and all were taking medication that had the potential to cause dry mouth. This may present an opportunity for clinical pharmacists to prioritise a more “at risk” group.

In three-quarters of cases where there was difficulty, we found that there was an alternative formulation available (or one in the same drug class). However, it should be acknowledged that liquid preparations may not always be the optimal alternative [53], and a patient specific approach should be considered.

Ideally patients should complete the SWAMECO at admission and these data can then be reviewed by the ward clinical pharmacist, in conjunction with other healthcare colleagues. Patients can then be assessed and receive specific and clear advice on swallowing strategies. In addition, alternative formulations may be available that would improve the patients experience as well as medication adherence.

### Further research

Further research is required to establish whether SWAMECO could feasibly form part of the admission process and to clarify whether the SWAMECO data adds value to the care of these patients.

## Conclusion

The SWAMECO provides valuable and timely information on an individual’s ability to swallow medicines. This will offer clinical pharmacists the opportunity to intervene and provide expert medication advice to their prescribing colleagues and patients. As patients may modify, or indeed skip, their medicines, without the guidance of healthcare professionals, it is crucial that we adopt a proactive approach to facilitate both the safe administration of medicines and medication adherence.

## Supplementary Information

Below is the link to the electronic supplementary material.

Supplementary file1 (DOCX 14 KB)
