# Supplementary material for: Application of a screening tool to understand the medication habits of patients with swallowing difficulty: a prospective observational study
Source: Int J Clin Pharm. 2025 Mar 27;47(5):1224–31. doi: 10.1007/s11096-025-01901-7 (PMC12432030; doi:10.1007/s11096-025-01901-7)
Supplement: Supplementary file 1 — Supplementary file1 (DOCX 14 KB) [file 11096_2025_1901_MOESM1_ESM.docx]

**Supplemental table 1** Patients self-reporting of how much their swallowing difficulty affected them (Item 7).

| **Rating scale** | **Number of patients n = 14** |
| --- | --- |
| **0** | 0 |
| **1** | 0 |
| **2** | 1 |
| **3** | 2 |
| **4** | 0 |
| **5** | 3 |
| **6** | 2 |
| **7** | 2 |
| **8** | 3 |
| **9** | 0 |
| **10** | 1 |

**Supplemental Table 2** Medicines reported by patients as difficult to swallow (Item 8)

| **Frequency of report** | **Medication name** |
| --- | --- |
| **6** | Paracetamol |
| **5** | Large medication |
| **3** | Small medication, chalky medication or no FC |
| **1** | Thiamine, Magnesium, Asacolon® 800mg, antibiotic tablets, aspirin, steroids, rifaximin, cholestagel®, serc®, zopiclone, Ponstan®, evening primrose oil, Ranexa®, lyrica®, oxynorm®, flurazepam, large chemotherapy, halved Aldactone®, all medication, capsules, quetiapine. |

Supplemental Table 3 **Description of feeling reported by patients when swallowing medicines**

| **Description of feeling reported by patients when swallowing medicines** | **Number of times a patient reported the feeling when medicines are swallowed (n = 14)** |
| --- | --- |
| **Choking** | 5 |
| **Having a fit of coughing** | 8 |
| **Suffocation** | 4 |
| **Going to Gag or be sick** | 6 |
| **Medicine(s) gets stuck** | 12 |
| **pain** | 5 |
| **Other (free text)** |  |
| Discomfort | 3 |
| Feels medicine in throat | 2 |
| Feels medicine breaks-up in throat | 1 |
| Voice change | 1 |
| **Total** | 47 |
|  |  |
